# Supplementary material for: An efficient and precise solution-vacuum hybrid batch fabrication of 2D/3D perovskite submodules
Source: Nat Commun. 2025 Jul 31;16:7019. doi: 10.1038/s41467-025-62392-8 (PMC12314120; doi:10.1038/s41467-025-62392-8)
Supplement: Supplementary file 1 — Supplementary Information [file 41467_2025_62392_MOESM1_ESM.pdf]

Supplementary Information

An Efficient and Precise Solution-Vacuum Hybrid Batch Fabrication of 2D/3D Perovskite Submodules

Yingping Fan[1,2,#], Zhixiao Qin[3,#], Lei Lu[1], Ni Zhang[1], Yugang Liang[1], Shaowei Wang[1], Wenji Zhan[1], Jiahao Guo[1], Haifei Wang[1], Yuetian Chen[1,4*], Yanfeng Miao[1,4*] & Yixin Zhao[1,2,4*]

1. School of Environmental Science and Engineering, Frontiers Science Center for Transformative Molecules, State Key Laboratory of Green Papermaking and Resource Recycling, Shanghai Jiao Tong University, Shanghai 200240, China.
2. Future Photovoltaic Research Center, Global Institute of Future Technology, Shanghai Jiao Tong University (SJTU-GIFT), Shanghai 200240, China.
3. Shanghai Pvsktech Co., Ltd., Shanghai 201109, China.
4. Shanghai Non-carbon Energy Conversion and Utilization Institute, Shanghai 200240, China.

#. These authors contributed equally: Yingping Fan, Zhixiao Qin

* Corresponding authors. Emails: yixin.zhao@sjtu.edu.cn, yanfengmiao@sjtu.edu.cn, yuetian.chen@sjtu.edu.cn.

Supplementary Figs 1-31

Supplementary Tables 1-4

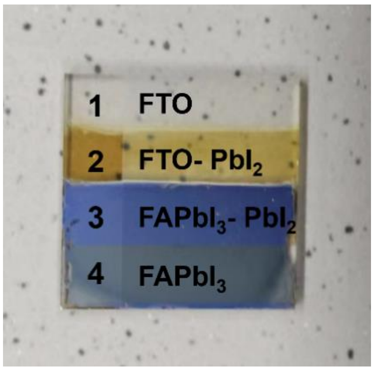

Supplementary Fig. 1 Photo image of the perovskite film after PbI_2 evaporation.

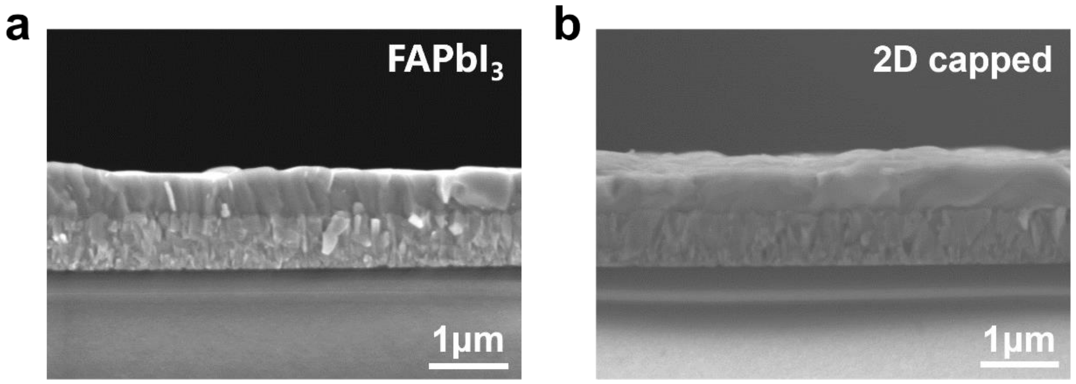

Supplementary Fig. 2 Cross-sectional SEM image of (a)$FAPbI_3$ and (b) 2D capped sample.

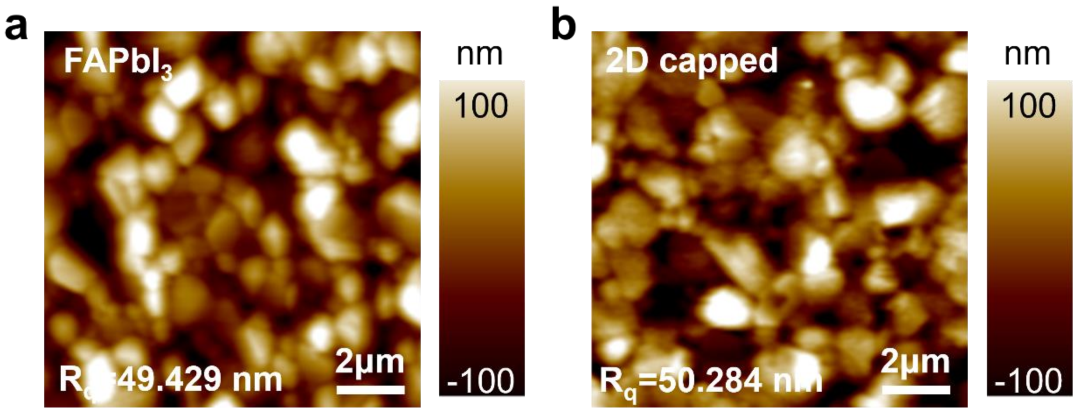

Supplementary Fig. 3 AFM images of (a) $FAPbI_3$ and (b) 2D capped samples.

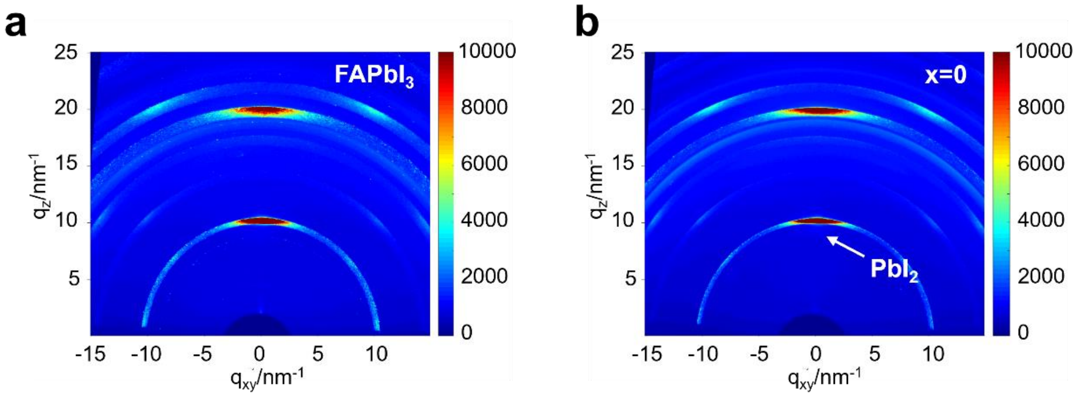

Supplementary Fig. 4 GIWAXS patterns of (a) $FAPbI_3$ and (b) x=0 samples.

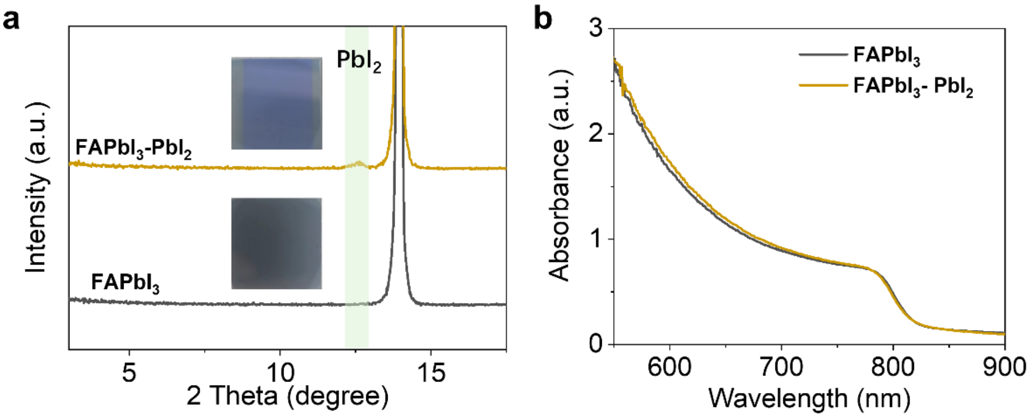

Supplementary Fig. 5 (a) XRD patterns of perovskite films without or with PbI_2. The inset photo images are the corresponding films after PbI_2 evaporation. (b) UV-vis absorption spectra of perovskite films without or with PbI_2.

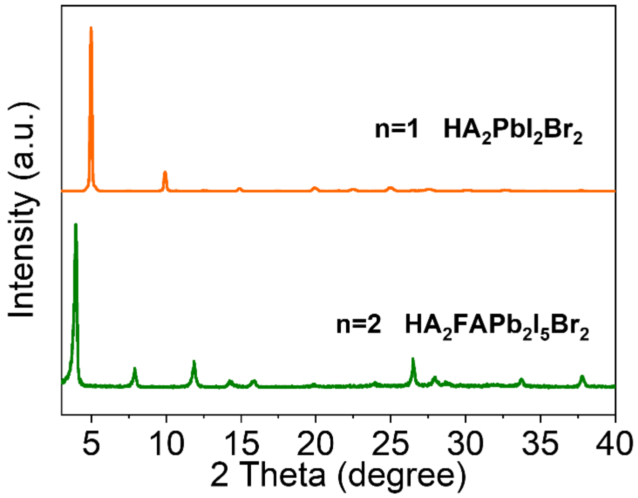

Supplementary Fig. 6 XRD patterns of $HA_2PbI_2Br_2$ (n=1) and $HA_2FAPbI_5Br_2$ (n=2) perovskite films.

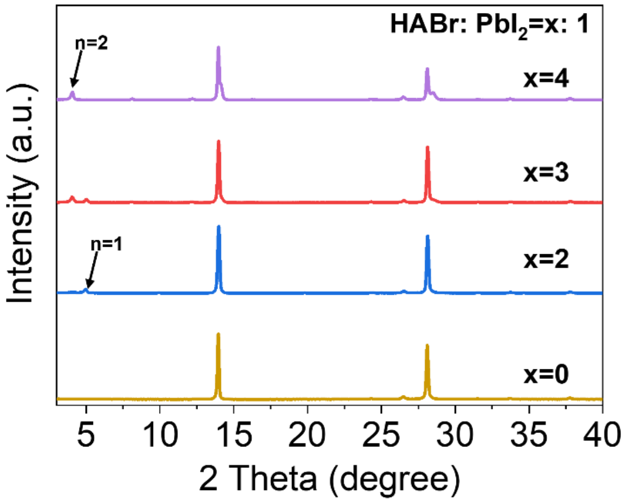

Supplementary Fig. 7 XRD patterns of perovskite films with HABr: PbI_2 =x:1, x is 0, 2, 3 and 4, respectively.

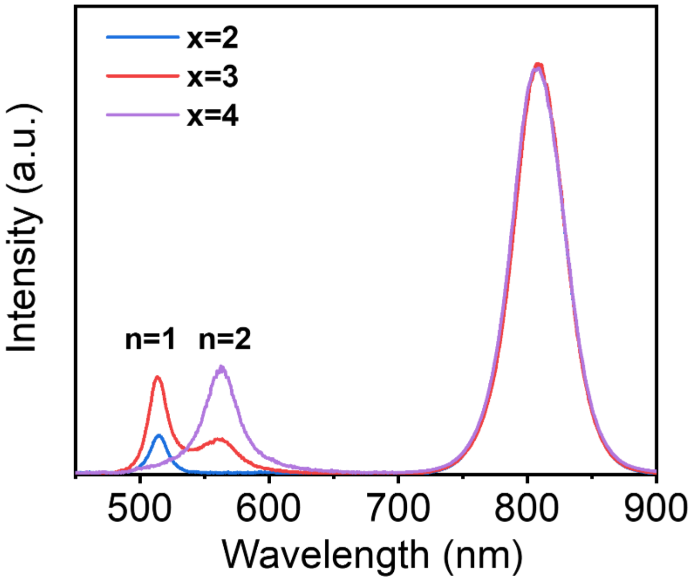

Supplementary Fig. 8 PL spectra of $FAPbI_3$ perovskite films with HABr: PbI_2 =x:1, x is 2, 3 and 4, respectively.

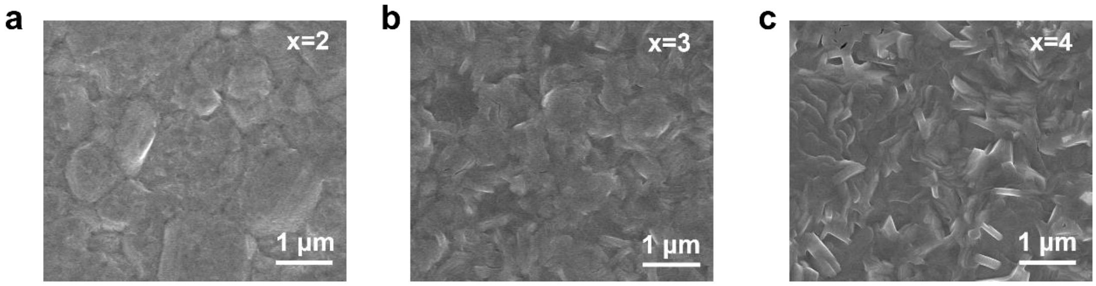

Supplementary Fig. 9 Top-view SEM images of $FAPbI_3$ films with different molar ratio of HABr to PbI_2, (a) x=2, (b) x=3 and (c) x=4, respectively.

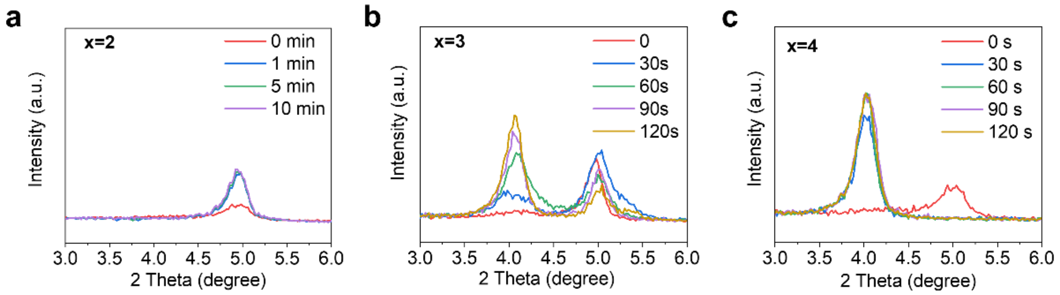

Supplementary Fig. 10 XRD patterns of (a) x=2, (b) x=3 and (c) x=4 samples with different annealing times.

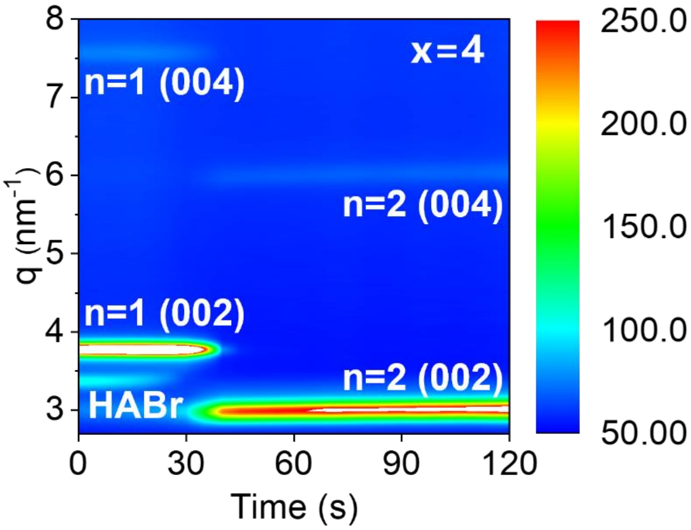

Supplementary Fig. 11 *in-situ* GIWAXS patterns of x=4 sample.

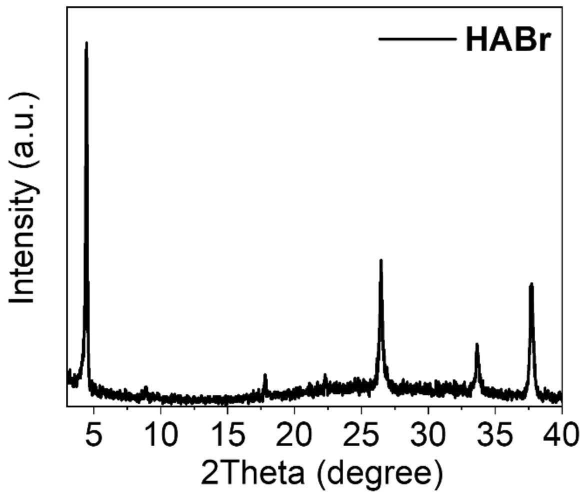

Supplementary Fig. 12 XRD pattern of HABr.

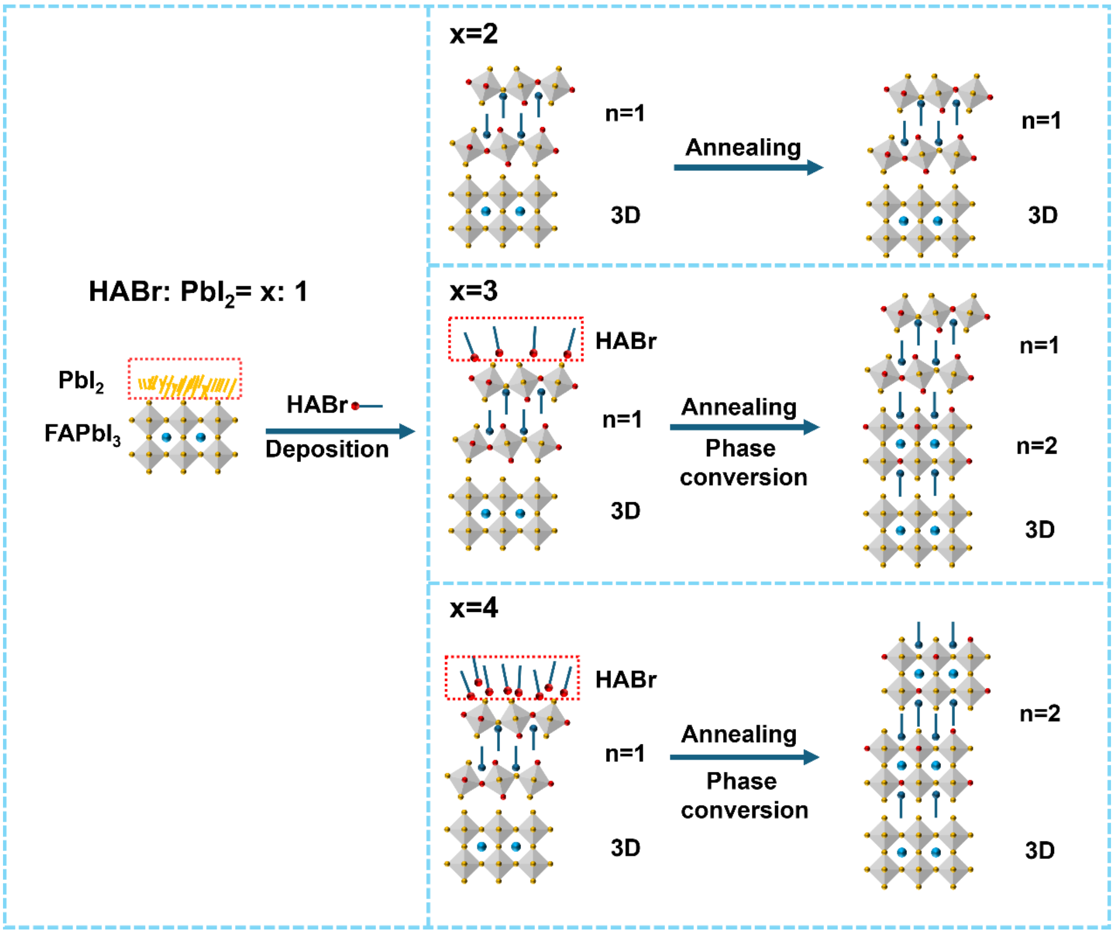

Supplementary Fig. 13 Mechanism schematic diagram of the formation and conversion process of the 2D perovskites capping layer with different molar ratio of HABr to PbI_2.

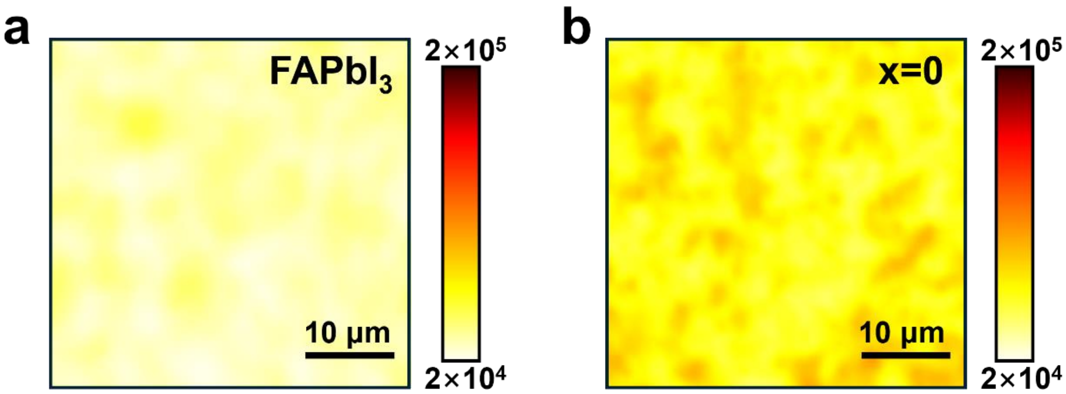

Supplementary Fig. 14 PL mapping images of (a) $FAPbI_3$ and (b) x=0 samples.

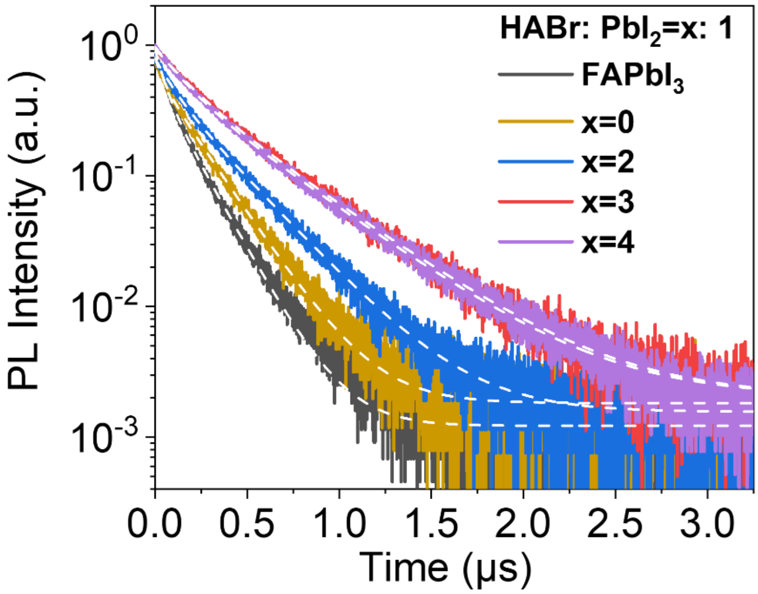

Supplementary Fig. 15 TRPL spectra of $FAPbI_3$ perovskite films with HABr: PbI_2 =x:1, x is 0, 2, 3 and 4, respectively.

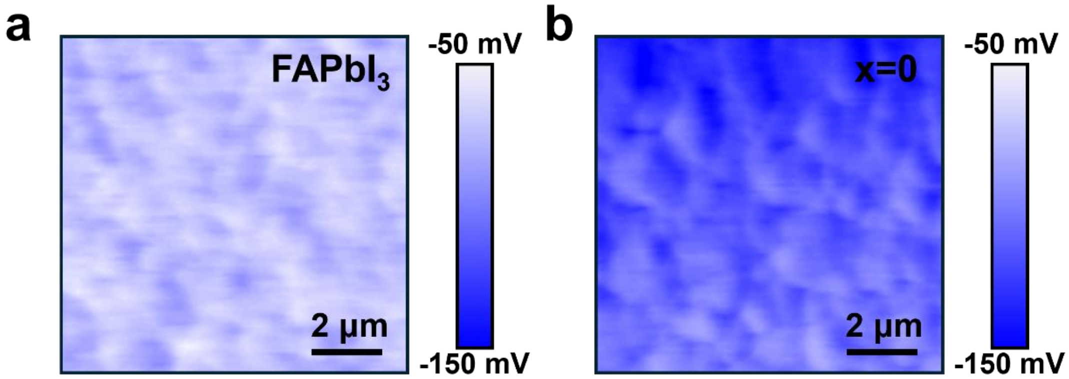

Supplementary Fig. 16 KPFM images of (a) $FAPbI_3$ and (b) x=0 samples.

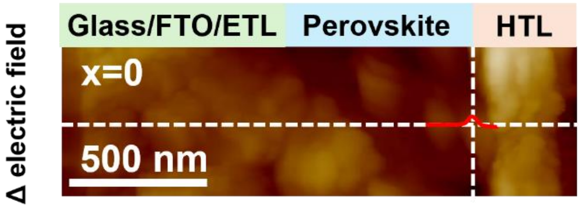

Supplementary Fig. 17 Cross-section of KPFM images of x=0 samples.

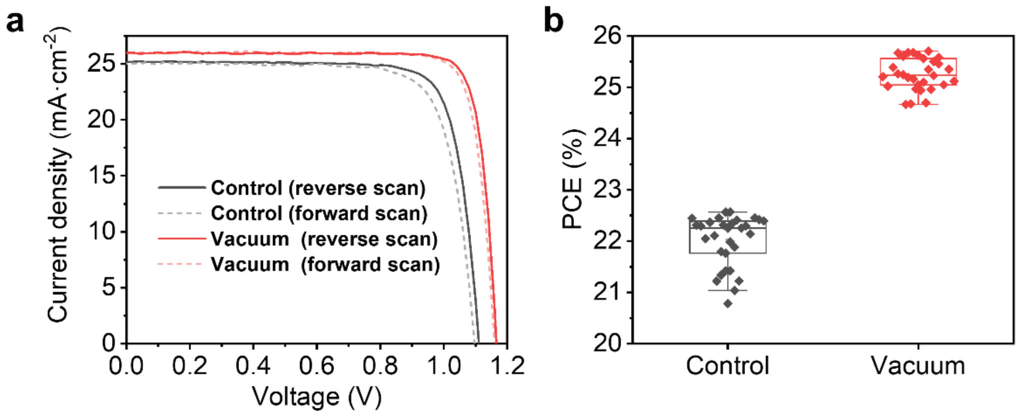

Supplementary Fig. 18 (a) *J*-*V* curves of the control and vacuum PSCs under both reverse and forward scans. (b) The PCE statistical parameters of 30 control and vacuum PSCs. The box plots show the 25–75% box limits and the median. The whiskers show the 1.5 interquartile range.

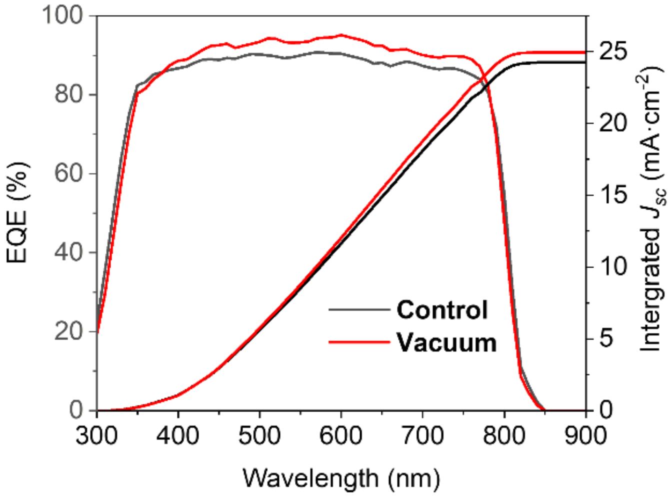

Supplementary Fig. 19 EQE and the integrated J_{sc} of the control and vacuum PSCs.

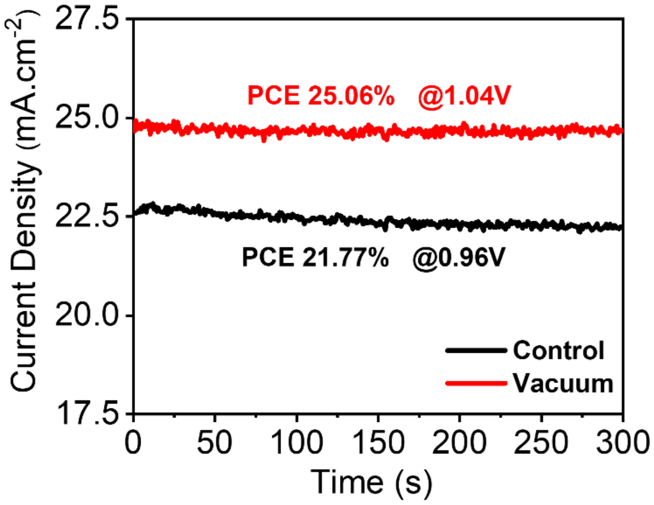

Supplementary Fig. 20 Steady-state output test of the control and vacuum devices.

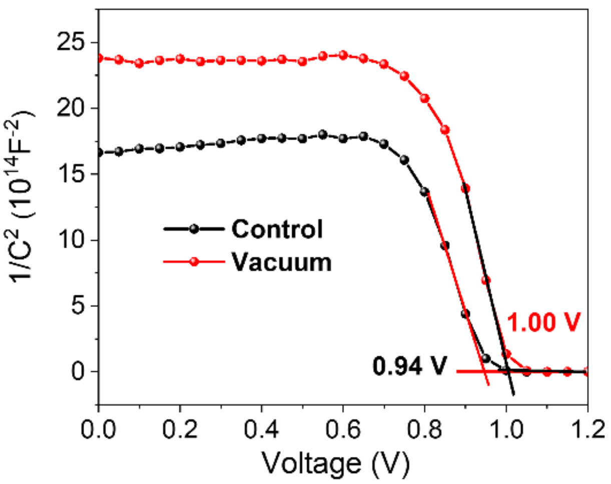

Supplementary Fig. 21 Mott-Schottky plots of the control and vacuum devices.

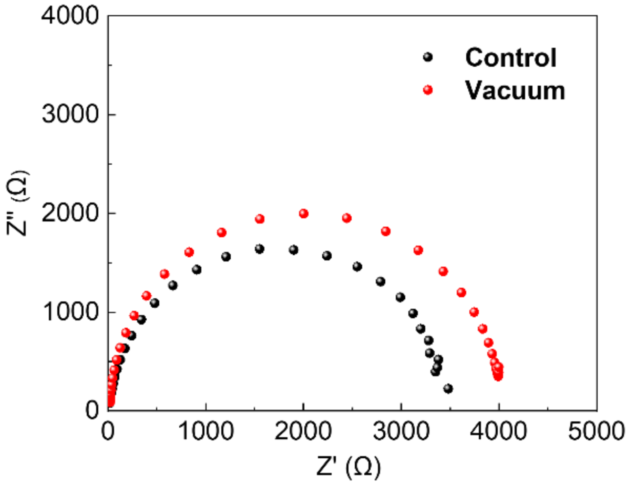

Supplementary Fig. 22 EIS spectra of control and vacuum devices.

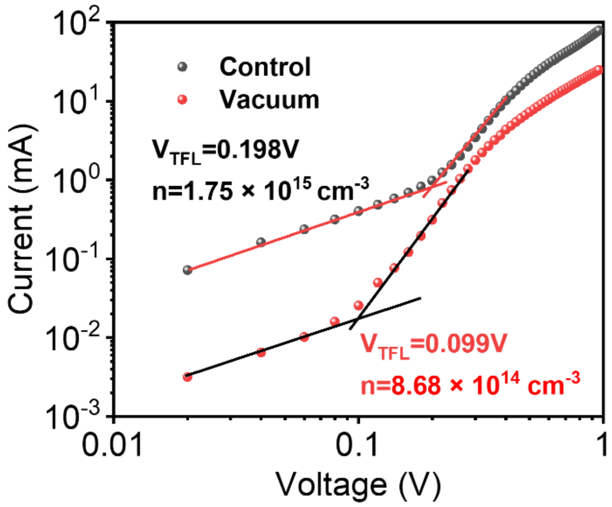

Supplementary Fig. 23 The trap-filled limiting voltage in the space-charge limited current (SCLC) measurements of the control and vacuum films.

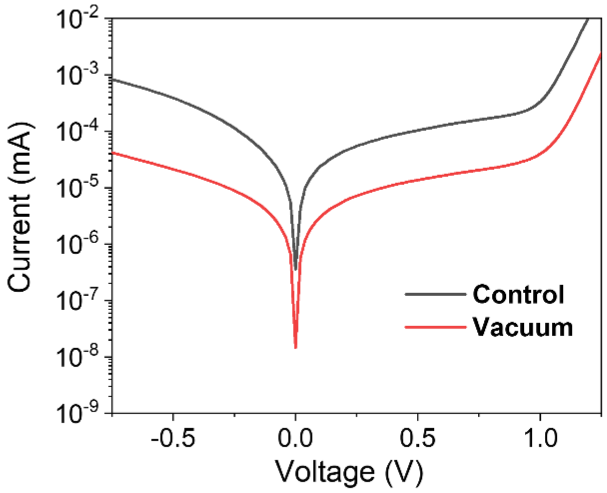

Supplementary Fig. 24 Dark current measurements of the control and vacuum PSCs.

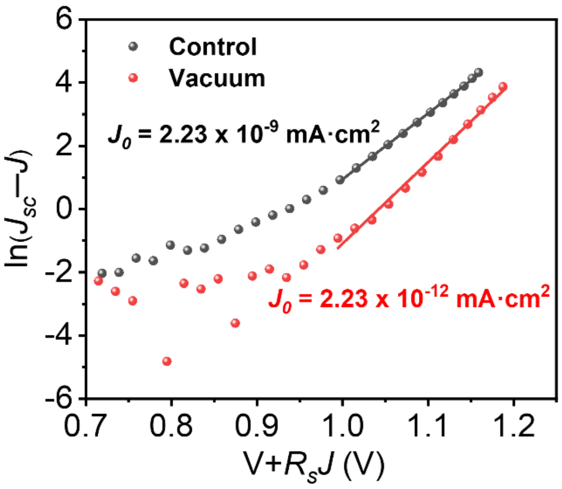

Supplementary Fig. 25 plot of ln (J_{sc}–J) with respect to V + R_sJ.

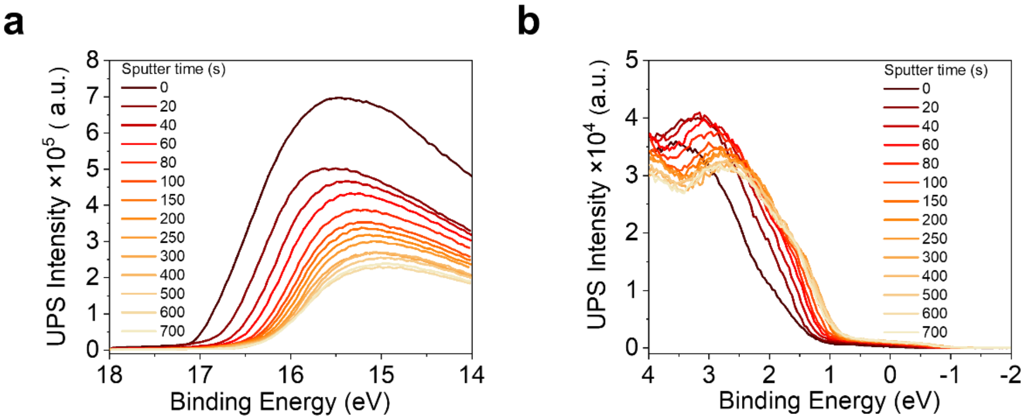

Supplementary Fig. 26 Depth-dependent ultraviolet photoemission spectroscopy (UPS) (a) at the secondary cut off energy for vacuum sample. (b) at the valence band maximum for vacuum sample.

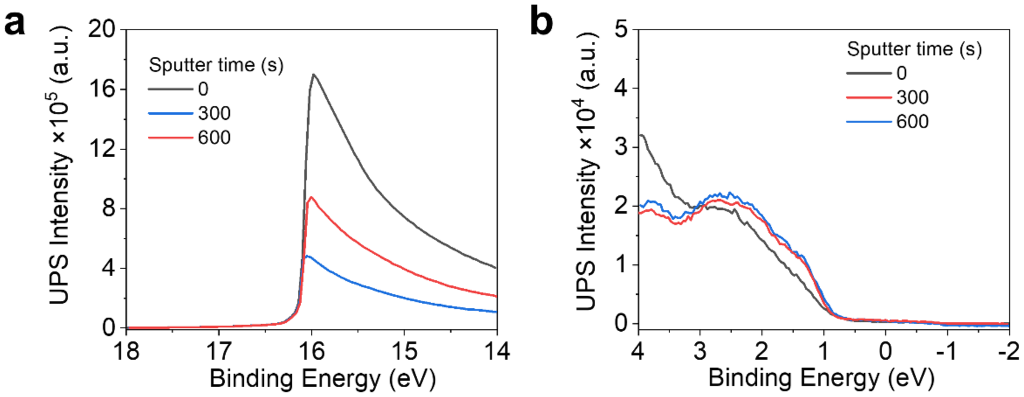

Supplementary Fig. 27 Depth-dependent ultraviolet photoemission spectroscopy (UPS) (a) at the secondary cut off energy for control sample. (b) at the valence band maximum for control sample.

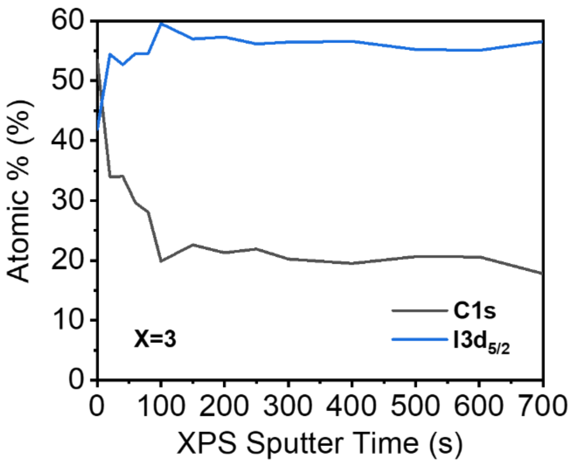

Supplementary Fig. 28 Atomic percentage determined from depth-dependent XPS of vacuum sample.

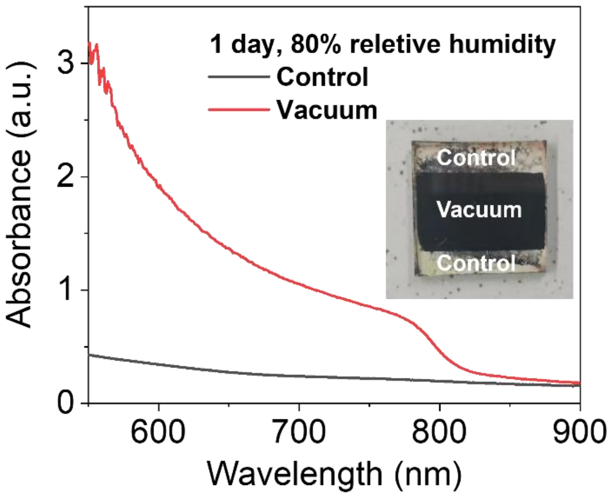

Supplementary Fig. 29 Moisture stability of control and vacuum samples when stored under 80% RH at 25°C in ambient air for 1 day. The inset image is $FAPbI_3$ perovskite thin films with and without a 2D capping layer after being stored under 80% RH at 25°C in ambient air for 1 day.

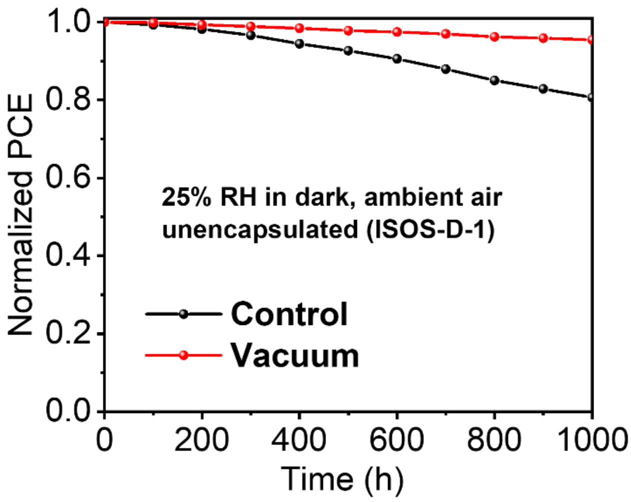

Supplementary Fig. 30 The shelf-life stability of unencapsulated control and vacuum PSCs.

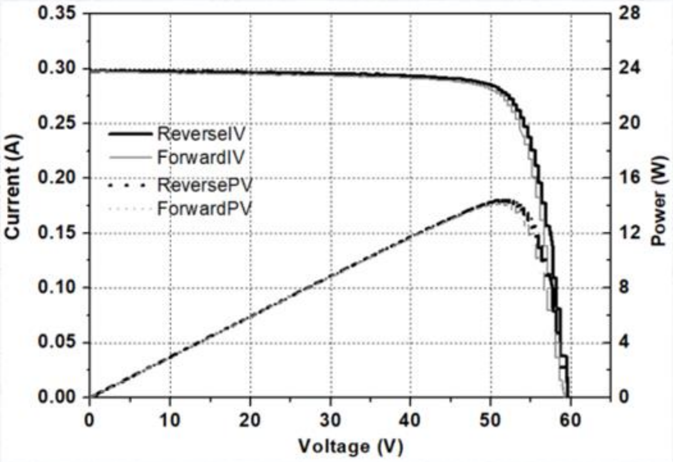

图 1 钙钛矿层样品 I-V 曲线图 Perovskite I-V Characteristic curves

电池类型 Type	扫描方向 Scaning Direction	指定面积 (m^2)	短路电流 I_{sc}(A)	开路电压 V_{oc}(V)
钙钛矿 Perovskite	反扫 Reverse	0.0663	0.300	59.64
最大功率 P_{max}(W)	最大功率电流 I_{max}(A)	最大功率电压 V_{max}(V)	填充因子 FF (%)	转换效率 η (%)
14.45	0.276	51.90	80.83	21.79

Supplementary Fig. 31 Certification report of the 30×30 cm^2 submodule, measured at The National Institute of Metrology.

Supplementary Table 1 Fitting parameters for TRPL spectra of perovskite film samples.

	τ_{ave} (ns)	τ_1 (ns)	A_1	τ_2 (ns)	A_2
$FAPbI_3$	147.09	48.48	0.41	169.47	0.52
x=0	194.04	42.15	0.21	205.41	0.59
x=2	263.87	96.48	0.27	291.58	0.53
x=3	388.05	167.48	0.42	447.27	0.56
x=4	384.86	146.05	0.38	439.99	0.57

Supplementary Table 2 Parameters of $FAPbI_3$ perovskite devices with HABr: PbI_2 =x:1, x is 0, 2, 3 and 4, respectively.

	V_{oc} (V)	J_{sc} (mA·cm^{-2})	*FF* (%)	PCE (%)
$FAPbI_3$	1.110	25.17	80.34	22.45
x=2	1.156	26.07	81.87	24.67
x=3	1.165	25.98	84.92	25.70
x=4	1.038	20.59	64.74	13.83

Supplementary Table 3 Corresponding parameters of vacuum sample calculated from depth-dependent UPS measurements.

**Etching time (s)**	**VB (eV)**	**E_{cutoff} (eV)**	**WF (eV)**	**VBM (eV)**
0	1.3	17	-4.22	-5.52
20	1.27	16.72	-4.5	-5.77
40	1.2	16.56	-4.66	-5.86
60	1.13	16.45	-4.77	-5.9
80	1.07	16.36	-4.86	-5.93
100	1	16.32	-4.9	-5.9
150	0.9	16.26	-4.96	-5.96
200	0.89	16.2	-5.02	-5.91
250	0.88	16.14	-5.08	-5.96
300	0.85	16.14	-5.08	-5.93
400	0.86	16.1	-5.12	-5.98
500	0.86	16.09	-5.13	-5.99
600	0.87	16.12	-5.1	-5.97
700	0.9	16.13	-5.09	-5.99

Supplementary Table 4 Corresponding parameters of control sample calculated from depth-dependent UPS measurements.

**Etching time (s)**	**VB (eV)**	**E_{cutoff} (eV)**	**WF (eV)**	**VBM (eV)**
0	0.92	16.14	-5.08	-6.00
300	0.88	16.13	-5.09	-5.97
600	0.88	16.14	-5.08	-5.96
