# Supplementary material for: An efficient and precise solution-vacuum hybrid batch fabrication of 2D/3D perovskite submodules
Source: Nat Commun. 2025 Jul 31;16:7019. doi: 10.1038/s41467-025-62392-8 (PMC12314120; doi:10.1038/s41467-025-62392-8)
Supplement: Supplementary file 2 — Reporting summary [file 41467_2025_62392_MOESM2_ESM.pdf]

nature portfolio

Corresponding author(s): Yixin Zhao, Yanfeng Miao, Yuetian Chen

# Solar Cells Reporting Summary

Nature Portfolio wishes to improve the reproducibility of the work that we publish. This form is intended for publication with all accepted papers reporting the characterization of photovoltaic devices and provides structure for consistency and transparency in reporting. Some list items might not apply to an individual manuscript, but all fields must be completed for clarity.

For further information on Nature Research policies, including our data availability policy, see Authors & Referees.

## Experimental design

**Please check the following details are reported in the manuscript, and provide a brief description or explanation where applicable.**

### 1. Dimensions

Area of the tested solar cells
- [x] Yes
- [ ] No

The active area of perovskite solar cells is 0.088 cm2 and the aperture area of perovskite solar modules is 663 cm2

*Explain why this information is not reported/not relevant.*

Method used to determine the device area
- [x] Yes
- [ ] No

The aperture area of PSMs can be found in Supplementary Fig.31 the certification report of the 30×30 cm2 submodule, measured at The National Institute of Metrology.

*Explain why this information is not reported/not relevant.*

### 2. Current-voltage characterization

Current density-voltage (J-V) plots in both forward and backward direction
- [x] Yes
- [ ] No

Current density-voltage (J-V) plots in both forward and backward direction can be found in Supplementary Fig. 18.

Voltage scan conditions
- [x] Yes
- [ ] No

J-V curves of perovskite solar cells were measured under an inert atmosphere by a Keithley 2401 source meter with a scan rate of 20 mV s−1 under simulated AM 1.5 G illumination (100 mW cm−2; Enlitech Class AAA Solar Simulator).

*Explain why this information is not reported/not relevant.*

Test environment
- [x] Yes
- [ ] No

J-V curves were measured in a N2-filled glove box.

*Explain why this information is not reported/not relevant.*

Protocol for preconditioning of the device before its characterization
- [x] Yes
- [ ] No

Devices were stored in a dry-air glove box.

*Explain why this information is not reported/not relevant.*

Stability of the J-V characteristic
- [x] Yes
- [ ] No

MPP tracking was measured under ambient air condition and a temperature of 65 ̊C (ISOS-L-2).

*Explain why this information is not reported/not relevant.*

### 3. Hysteresis or any other unusual behaviour

Description of the unusual behaviour observed during the characterization
- [ ] Yes
- [x] No

*Provide a description of hysteresis or any other unusual behaviour observed during the characterization.*

There was no unusual behavior observed during the characterization.

Related experimental data
- [ ] Yes
- [x] No

*Provide a description of the related experimental data.*

There was no unusual behavior observed during the characterization.

### 4. Efficiency

External quantum efficiency (EQE) or incident photons to current efficiency (IPCE)
- [x] Yes
- [ ] No

Supplementary Fig. 19

*Explain why this information is not reported/not relevant.*

A comparison between the integrated response under the standard reference spectrum and the response measure under the simulator

☐ Yes
☐ No

External quantum efficiency (EQE) spectra and integrated current density in Supplementary Fig. 17 matched well with the measured Jsc.

*Explain why this information is not reported/not relevant.*

For tandem solar cells, the bias illumination and bias voltage used for each subcell

☐ Yes
☒ No

*Provide a description of the measurement conditions.*

No tandem solar cells.

5. Calibration

Light source and reference cell or sensor used for the characterization

☒ Yes
☐ No

The light source is Enlitech Class AAA Solar Simulator.

*Explain why this information is not reported/not relevant.*

Confirmation that the reference cell was calibrated and certified

☒ Yes
☐ No

We calibrated the intensity of the light source using a standard Si solar cell from a certified laboratory.

*Explain why this information is not reported/not relevant.*

Calculation of spectral mismatch between the reference cell and the devices under test

☐ Yes
☒ No

*Provide a value of the spectral mismatch and/or a description of how it has been taken into account in the measurements.*

The light spectrum matches well with the reference Si solar cell.

6. Mask/aperture

Size of the mask/aperture used during testing

☒ Yes
☐ No

The size of the mask is 0.088 cm2.

*Explain why this information is not reported/not relevant.*

Variation of the measured short-circuit current density with the mask/aperture area

☐ Yes
☐ No

The mask size was not varied.

*Explain why this information is not reported/not relevant.*

7. Performance certification

Identity of the independent certification laboratory that confirmed the photovoltaic performance

☒ Yes
☐ No

The photovoltaic performance of perovskite solar module was certified by the The National Institute of Metrology.

*Explain why this information is not reported/not relevant.*

A copy of any certificate(s)

☒ Yes
☐ No

The certificate copy was provided in Supplementary Fig. 31.

*Explain why this information is not reported/not relevant.*

8. Statistics

Number of solar cells tested

☒ Yes
☐ No

30 solar cells for each condition.

*Explain why this information is not reported/not relevant.*

Statistical analysis of the device performance

☒ Yes
☐ No

Supplementary Fig. 18b

*Explain why this information is not reported/not relevant.*

9. Long-term stability analysis

Type of analysis, bias conditions and environmental conditions

☒ Yes
☐ No

The operational stability of the perovskite solar cells was investigated by aging the encapsulated devices under ambient air condition and using MPP tracking under a temperature of 65 ℃ (ISOS-L-2).

*Explain why this information is not reported/not relevant.*
